# Trends in prescription and cost of Sativex, a cannabinoid-based medicine, in treating patients with multiple sclerosis in England

**DOI:** 10.1080/20523211.2024.2342318

**Published:** 2024-05-08

**Authors:** Farideh A. Javid, Anam Alam, Emily Williams, Sidhra Sajid Malik, Usama Mohayuddin, Syed Shahzad Hasan

**Affiliations:** Department of Pharmacy, School of Applied Sciences, University of Huddersfield, Huddersfield, UK

**Keywords:** Sativex, nabiximols, MS, spasticity, and pain

## Abstract

**Aim:**

Cannabis-based medication has recently been made available in the NHS for reducing pain and spasticity in patients with multiple sclerosis (MS). The currently available preparation of Sativex (nabiximols) contains a combination of botanical cannabis extracts with cannabidiol (CBD) and tetrahydrocannabinol (THC) with almost equal amounts in addition to minor cannabinoids and terpenoids and is delivered via an oro-mucosal spray. The present study aims to examine the use and trends in prescribing cannabinoid-based Sativex to control pain in patients diagnosed with MS.

**Methods:**

Primary care prescribing data for cannabinoid-based Sativex (2013-2022) from the Prescription Cost Analysis were extracted and analysed. Linear regression analyses were performed to examine prescription trends and prescription costs (average change per year).

**Results:**

There was a general increasing trend in the number of prescriptions each year, from 4.42 items dispensed per 100,000 people in 2013 to 5.15 in 2022. Overall, prescription items for cannabinoid-based Sativex increased by 0.34% per year (95% CI:−3.98, 4.67, *p* = 0.860) on average between 2013 and 2022. On average, a 2.43% (95% CI: −5.78, 0.92, *p* = 0.133) increase per year was observed for the costs of cannabinoid-based Sativex from 2013 to 2022.

**Conclusion:**

The results suggested that cannabinoid-based Sativex should be considered an option due to its effectiveness, acceptable tolerance, and safety profile in the prescribing of Sativex.

## Introduction

Multiple sclerosis (MS) is an autoimmune neurodegenerative disease in which inflammation, demyelination, and axonal damage are classic characteristics (Ward & Goldman, 2022). The incidence and prevalence of MS peak at approximately 30 and 50 years of age, respectively (Koch-Henriksen & Sørensen, 2010). The prevalence of MS in England is 190 cases per 100,000 population (Public Health England, 2020). Treatment options aim to treat relapses, reduce the number of relapses, and treat specific symptoms. The use of steroids such as oral methylprednisolone as first-line treatment is recommended to manage relapse (NHS, 2018). Disease-modifying drugs are also used as a treatment option in patients with active relapsing-remitting multiple sclerosis as they reduce the number of relapses (NICE, 2019).

50–80% of patients with MS report persistent pain. Patients describe pain as central neuropathic pain, back pain, painful tonic spasms, and headache. Central neuropathic pain is successfully treated with antidepressants and anticonvulsants (Thibault et al., 2012). If these recommended pharmacological treatments do not manage spasticity, NICE guidelines suggest offering a 4-week trial of a cannabis-based THC: CBD spray (Sativex) to treat moderate to severe spasticity (NICE, 2021).

Sativex is a botanical mixture containing primarily delta-9-tetrahydrocannabinol and cannabidiol along with minor cannabinoids and terpenoids from *Cannabis sativa* L. that has been accepted as a unitary formulation by regulatory bodies in 30 countries (Electronic Medicines Compendium, 2015, 2019). Sativex is the first and only licenced cannabinoid-based medication prescribed for spasticity associated with MS in the UK since 2010 (Electronic Medicines Compendium, 2019), and a Cochran review in 2022 focused on the medicinal use of Sativex (Cochran, 2022). Recent clinical studies have indicated that adding Sativex to the patient’s medication list provided a significant improvement in resistant MS spasticity compared with the first-line anti-spasticity medication alone (Rekand, 2014; Rog et al., 2005; Markovà et al., 2019).

Although the accumulated data indicates that cannabinoid-based Sativex is well tolerated (Serpell et al., 2013, Rekand, 2014), a recent study focused on the perception of cannabis-based medications amongst healthcare professionals across Europe indicated that many healthcare professionals may not have enough knowledge of cannabinoid-based medications such as Sativex, when it comes to prescribing. This recent study consequently suggests a low rate of prescriptions (Jouanjus et al., 2023). Therefore, this study attempted to investigate trends in the prescribing and cost of cannabinoid-based Sativex. The objective was to examine if there was increased uptake (in the number of prescriptions and costs) of cannabinoid-based Sativex in primary care England.

## Methods

This study examines the prescribing trends and costs of cannabinoid-based Sativex in primary care England, to understand any consequent changes in use and NHS prescription reimbursements. Prescribing data for cannabinoid-based Sativex between January 2013 and December 2022 was obtained from publically available Prescription Cost Analysis data (PCA). These data highlight the quantities of each drug unit, prescription items dispensed, and net ingredient costs from all regions of England, allowing for the analysis of prescription trends and costs.

Prescription Cost Analysis (PCA) is a primary care database that maintains the details of the number of prescription items and costs of all NHS prescriptions dispensed in the community (NHS BSA, 2020). A prescription item is a single drug/ medicine prescribed by doctors, dentists, or even non-medical prescribers such as nurses and pharmacists. For example, if cannabinoid-based Sativex were prescribed, it would count as one item dispensed, regardless of how many packets or tablets were supplied. However, if different strengths of the same medicine are prescribed, it would count as two dispensed items.

The quantity of drug depends on the product's formulation and is measured in units (tablet, capsule, ml). Net ingredient cost is the price of the medicine. i.e. the price listed in Part VIII or XI of the drug tariffs or for drugs not listed in the drug tariff, the listed price published by the manufacturer, wholesaler, or supplier. The drug costs before discounts are applied do not include dispensing costs or fees. The PCA data from the NHS BSA database were collected for each month from 2013 to 2022, which allowed a review of the trend of Sativex.

Prescription cost analysis (PCA) data for Sativex were extracted using the NHS BSA website to compare prescribing and cost trends to be made. Data from the pharmacy, appliance contractor, and prescribing doctors were combined to extract the quantity and items dispensed and the net ingredient cost from 2013 to 2022. The total quantities for the number of itemsitems, the quantity of items, and the NIC were calculated and summed to find the total quantity of items dispensed per year in units of a thousand. The prescription items were also presented as items dispensed per 100,000 general population. This data was extracted and imported into Microsoft Excel to produce figures.

For cost analysis, inflation-adjusted costs were used for the year 2022 compared to the year 2013; this adjustment was calculated using The Bank of England inflation calculator (Bank of England, 2021).

Linear regression analysis was used to find the average percentage change per year, using the year as the independent variable and the prescription items dispensed / costs as the dependent variable. The percentage average change per year was obtained by dividing the regression coefficient by baseline prescriptions or costs, respectively, using the data from each year, starting from 2013.

SPSS software version 24 was used to conduct a statistical analysis. The regression analysis determines yearly trends in the number of prescriptions dispensed and the associated costs between 2013 and 2022.

## Results

**Figure 1.**
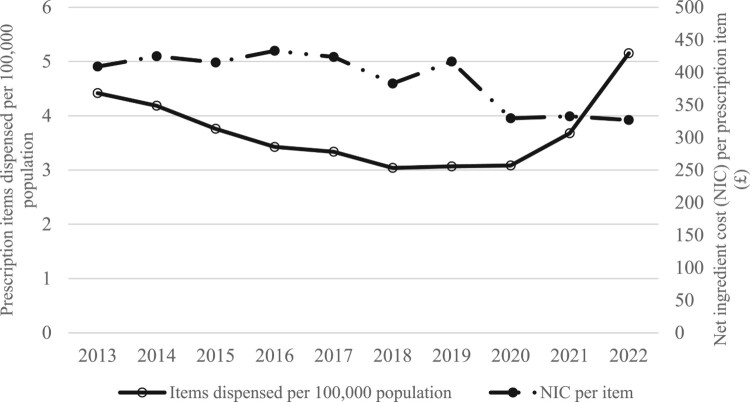
Trends of Sativex use (per 100,000 population) and costs in primary care England over the last decade (2013-2022).

There was a general increasing trend in the number of prescriptions of cannabinoid-based Sativex each year, from 4.42 items dispensed per 100,000 people in 2013 to 5.15 in 2022. However, between 2015 and 2018, there was a slight decrease in the number of prescriptions for cannabinoid-based Sativex each year. The results showed an increase in the number of prescription items of cannabinoid-based Sativex dispensed from 2013 to 2022 (Figure 1, Table 1). For example, in 2013, the number of cannabinoid-based Sativex items dispensed was 2,832, which increased to 3,483 items in 2022.

**Table 1.** The number of prescriptions dispensed per 100,000 population and inflation-adjusted costs.

Years	Number of prescription items dispensed	Items dispensed per 100,000 population	NIC per item (£)	Inflation-adjusted costs, £ (2022)
2013	2,832	4.42	408.94	505.23
2014	2,703	4.18	424.84	517.43
2015	2,448	3.76	415.18	505.05
2016	2,249	3.43	433.08	523.51
2017	2,204	3.34	423.72	499.23
2018	2,019	3.04	382.93	440.05
2019	2,049	3.07	416.61	470.69
2020	2,069	3.08	329.48	369.34
2021	2,477	3.68	332.50	363.30
2022	3,483	5.15	326.96	326.96

NIC = Net Ingredient Cost.

During 2013 and 2018, the number of prescription items distributed for Sativex slightly decreased, then in 2019, it started to increase, and finally, in 2022, it reached a higher level than in 2013, with the number of prescription items distributed reaching 3,483. Table 1 also shows the value for the items dispensed per 100,000 people, which in 2013 was 4.42, gradually decreasing to 3.04 in 2018. In 2019, the value started to increase slightly to 5.15 in 2022.

The inflation adjusted cost for 2013 (£505.23) shows the total cost spent on Sativex decreased in 2022 (£326.96) 1.5 times (Figure 1, Table 1). In 2013, the inflation-adjusted net ingredient cost of Sativex was over £500, which was reduced to £350 in 2022. The cost of Sativex stayed almost constant except for 2019 when the cost increased to £470.69 and then dropped to £326.96 in 2022.

A regression analysis of yearly trends in prescriptions dispensed and associated costs is shown in Table 2. In general, there was a 0.34 percent increase in prescriptions of Sativex per year (95% CI, −3.98, 4.67, *p* = 0.866) on average between 2013 and 2022. On average, an increase of 2.43% (95% CI: −5.78, 0.92, *p* = 0.133) per year was seen for the costs of Sativex from the year 2013 to 2022.

**Table 2.** Regression analysis of yearly trends in prescriptions and costs, 2013–2022.

Drugs	Prescription trends	
	Prescriptions, mean change per year as % of baseline^a^ (95% CI)	*p*
Prescription items	0.34 (−3.98, 4.67)	0.860
Net ingredient costs	−2.43 (−5.78, 0.92)	0.133

a = % change was calculated by dividing the regression coefficient by baseline prescriptions or costs from 2013 as given

## Discussion

The present study investigated the trends in prescribing Sativex. The results showed that the number of Sativex items prescribed increased from 2,832 in 2013 to 3,483 in 2022. The regression analysis showed an increase of 0.34% in Sativex items dispensed per year on average between 2013 and 2022. The data showed decreased prescription items dispensed from 2013 to 2018, followed by an upward trend. Sativex is used explicitly for neuropathic pain in MS patients, and its treatment can only be initiated by a specialist. The restricted use of this drug could be a factor contributing to a low number of prescriptions. However, due to the potential benefits it can bring to certain health conditions such as MS and the reassuring evidence regarding the safe use of Sativex, we expect prescribers to be more confident in prescribing this class of drugs to their patients in the future.

Currently, treating pain or spasticity in MS patients with Sativex (Oromucosal Spray) is expensive. It is prescribed on a private prescription that costs more than £500 for a month of supply; however, the NHS indicative price is £300 for the same dose (MS Scoeity UK, 2024). The findings are in line with previous studies carried out in the UK (Lu et al., 2012) and contrasted with the results obtained in European countries such as Italy, Spain, and Germany (Slof et al., 2015; Slof & Gras, 2012; Flachenecker, 2013). The governments of Spain and Germany reimbursed Sativex after the assessment that it was cost-effective. In fact, studies indicated that using Sativex was cost-effective and reduced physiotherapy and related costs in MS in Germany (Flachenecker, 2013). However, the high cost has not hindered UK clinicians from prescribing as the number of dispensed items has increased from 4.42 per 100,000 people in 2013 to 5.15 in 2022, reflecting the positive uptake of the medication.

## Current evidence on the efficacy and safety of Sativex

Overall, the outcomes from several randomised clinical trials were in favour of the use of cannabinoid-based Sativex for the management of neuromuscular disorders, particularly MS (Collin et al., 2007; Zajicek et al., 2012; Turri et al., 2018 Rekand et al., 2014; Rog et al., 2005). The main findings of the studies showed evidence that cannabinoid-based Sativex is significantly more effective in relieving pain associated with spasticity than placebo in patients with MS. Furthermore, these clinical trials also showed that cannabinoid-based Sativex was beneficial in alleviating pain and was also effective in improving mobility, sleep disturbances, and bladder control (Kavia et al., 2010; Bradley et al., 2004).

In a study in 2016, 21 MS patients whose symptoms have failed to respond to other treatments received cannabinoid-based Sativex. Once titrated, the average number of daily sprays was approximately 7, enough for the data to show a clinical benefit (Turri et al., 2018). Similar results were found in another study conducted in 2018 using 28 MS patients with chronic pain and spasticity. The patients were titrated using cannabinoid-based Sativex, and their daily number of sprays was recorded. Again, the mean number of daily sprays was approximately 7 and the results showed effective pain relief (Turri et al., 2018). The most common side effect of cannabinoid-based Sativex is dizziness, which is generally within the titration period of the first two weeks of treatment and is generally mild and will resolve with continued use after a few days (Nuara et al., 2016; Leocani et al., 2015). However, another study, which included 572 participants, showed no difference between the occurrence of adverse events between cannabinoid-based Sativex and the placebo group, which also experienced dizziness (Novotna et al., 2011).

A retrospective study on 396 MS patients for four years showed that following a four-week titration period, patients with milder physical symptoms and without cognitive impairment are more likely to benefit from the medication (Carotenuto et al., 2020). In line with the previous study, other studies also showed that patients with a shorter duration of the disease and milder symptoms after a titration phase were more likely to benefit and continue with the medication (Ferrè et al., 2016; Messina et al., 2017). The primary site of action for cannabinoid-based Sativex is the central nervous system. The lack of success in treatment in MS patients with severe symptoms could be that the source of the symptoms of muscular atrophy and contractures and fixed postures does not involve damage to the central nervous system (Pingel et al., 2017).

On the other hand, cognitive impairments such as long-term memory, attention, and speed of information processing have been reported in MS patients who also decline as physical disabilities progress (Lanzillo et al., 2016; Benedict et al., 2011). As a result, patients will have difficulty adapting and maintaining a strategy, for example, to deal with side effects such as bad mouth taste and complications in the oral cavity, and reducing tolerability. This will also lead to patients reporting a lack of efficacy and reducing the number of prescriptions. Therefore, cognitive impairment has been suggested to be a factor in lower treatment efficacy, reducing the number of prescriptions (Carotenuto et al., 2020).

Although the above studies suggested that Sativex will be successful if it begins early when milder symptoms are presented; however, other randomised controlled clinical trials showed that Sativex as an additional therapy had good efficacy in resistance patients with moderate to severe MS spasticity with mild to moderate side effects and without serious safety concerns (Markova et al., 2019). Therefore, the accumulated data indicate that reducing the number of prescriptions could involve reducing the efficacy of cannabinoid-based Sativex in some patients due to factors such as cognitive impairments and the advance in the stage of the disease, and prescription at the early stage of the disease is recommended.

## Strengths and limitations

This study adds important literature on the extent of Sativex prescribing in NHS England. The Prescription Cost Analysis database in this study offers population-level data on NHS primary care prescription and prescription costs in England. However, the data consist of prescriptions issued, and there are no details of the indications for the prescriptions, nor about the patients to whom the prescriptions are issued in terms of clinical or patient-related factors. Prescribing for other indications is not accounted for; it is mainly indicated as a treatment for symptom improvement in adult patients with moderate to severe spasticity due to multiple sclerosis. An increase in disease prevalence may explain some of the rising trends but it was unlikely to have a significant effect. Rising prescriptions do not necessarily indicate a rising number or proportion of medication users since long-term use and an increase in population size would also increase prescription numbers. The PCA data only cover prescriptions issued in primary care, and hence excludes drugs issued to patients in a hospital or on a private prescription.

## Conclusion

This study showed that the number of Sativex prescribed decreased from 2013 to 2018 and increased from 2019 to 2022. A recent surge in the number of prescriptions may indicate the treatment's effectiveness. It has become clear that many studies indicate the safety and tolerability associated with using Sativex; however, its high cost and restricted use may have reduced the number of prescriptions. Further research is required to examine its efficacy for a wider range of clinical indications.
